# Metagenome-assembled genome sequence of *Candidatus* Loosdrechtia sp. KJ reconstructed from an alkaline anammox reactor

**DOI:** 10.1128/mra.01024-25

**Published:** 2025-11-04

**Authors:** Han-Yin Chuang, Wei-Yu Chen, Shan-Hua Chen, Yung-Hsien Shao, Jer-Horng Wu

**Affiliations:** 1 Department of Environmental Engineering, National Cheng Kung University 34912, Tainan City, Taiwan; 2 Department of Environmental Engineering, National Ilan University 63371, Yilan, Taiwan; Rochester Institute of Technology, Rochester, New York, USA

**Keywords:** anammox, alkaline, nitrogen cycle

## Abstract

*Candidatus* Loosdrechtia sp. KJ is an anaerobic ammonium-oxidizing bacterium enriched from a bioreactor operated under alkaline conditions (pH 9.2 ± 0.4). We report its 3.39 Mb draft genome, containing 3,065 predicted coding sequences, 47 tRNA genes, and a single *rrn* operon.

## ANNOUNCEMENT

Anaerobic ammonium-oxidizing (anammox) bacteria are crucial contributors to the global nitrogen cycle and form a monophyletic lineage within the phylum *Planctomycetes*. Previous studies have demonstrated species-dependent ecological niche differentiation among anammox bacteria (1, 2). Most described anammox species proliferate optimally at pH 6.8–8.6 (2), while only a few reports document activity at higher alkaline conditions. Here, we present a draft metagenome-assembled genome of *Candidatus* Loosdrechtia sp. KJ recovered from an alkalㄍ anammox reactor operated at a pH of 9.2 ± 0.4 above the established optimal range.

*Ca*. Loosdrechtia sp. KJ was enriched from petrochemical wastewater sludge in a sequencing batch reactor fed with ammonium and nitrite (200–350 mg N/L each) as substrates and 500 mg/L potassium bicarbonate as the sole carbon source. The reactor conductivity was maintained at 12.4 ± 0.2 mS/cm. A sludge sample was harvested, filtered through a 0.22 µm sterilized mixed cellulose ester membrane (Whatman, UK), and used for genomic DNA extraction with the DNeasy PowerWater kit (Qiagen, Germany) following the manufacturer’s instructions. A DNA library was prepared with Enzymatic Fragmentation Kit 2.0 (Twist, USA) and sequenced on the Illumina NovaSeq 6000 platform (BIOTOOLS, Taiwan), yielding 2 × 150 bp paired-end reads. A total of 190,819,240 raw reads were generated. After trimming with Trimmomatic (v.0.39) (3) using the parameters ILLUMINACLIP:TruSeq3-PE.fa:2:30:10:2:True LEADING:12 TRAILING:12 SLIDINGWINDOW:4:15 MINLEN:36, 94,120,530 pairs of reads were retained. *De novo* assembly was conducted using Megahit (4) with the --presets meta-sensitive option. Binning was conducted using CONCOT (v.0.4.0) (5), MaxBin2 (v.2) (6), and MetaBAT2 (v.2.12.1) (7), followed by refinement with Metawrap (v.1.3.2) (8) using parameters -c 50 -x 10. Taxonomic assignment was performed using GTDB-Tk (v.2.4.0) (9) with the classify_wf workflow against GTDB release R220. The mean coverage was calculated using CoverM (v.0.6.1) (https://github.com/wwood/CoverM) with the -m mean option. Average nucleotide identity (ANI) and average amino acid identity (AAI) were calculated with FastANI (v.1.34) (10) and CompareM (v.0.1.2) (https://github.com/donovan-h-parks/CompareM), respectively. Default parameters were used except where otherwise noted.

The *Ca*. Loosdrechtia sp. KJ genome is 3,394,582 bp in length, assembled into 124 contigs with a GC content of 41.6%, an *N*_50_ of 60,255 bp, and 4,520× coverage. Genome completeness and contamination were estimated to be 98.9% and 1.65%, respectively, using CheckM (v.1.2.4) (11) with the lineage_wf workflow and -x fa extension. Functional annotation with Bakta (v.1.11.3) (12) identified 3,065 coding sequences, 47 tRNA genes, and a single *rrn* operon. Notably, DIAMOND Blastp (v.2.1.10) (13) searching against the National Center for Biotechnology Information nr database (v.2024.02) with an *e*-value cutoff of <10^−5^ detected all essential genes for anammox metabolism. Phylogenomic analysis placed *Ca*. Loosdrechtia sp. KJ in closest affiliation with *Ca*. Brocadiaceae bacterium WH-1 (GCA_030583665.1), sharing ANI and AAI values of 99.9%. Comparisons with *Ca*. Loosdrechtia aerotolerans (GCA_021646405.1), the only formally described anammox species of this genus (14), yielded ANI and AAI values of 77.9% and 75.0%, respectively. Although these values fall within the genus-level threshold (65%–95% for the same genus) (15), the relatively low ANI value indicates that *Ca*. Loosdrechtia harbors substantial genomic diversity.

## Data Availability

The whole-genome sequencing project for “*Ca*. Loosdrechtia sp. KJ” has been deposited in GenBank under accession number JBQWCT000000000. The raw sequencing reads have been deposited in the Sequence Read Archive under accession number SRR35197330.
